# The Association of Prenatal Exposure to Perfluorinated Chemicals with Glucocorticoid and Androgenic Hormones in Cord Blood Samples: The Hokkaido Study

**DOI:** 10.1289/EHP142

**Published:** 2016-05-24

**Authors:** Houman Goudarzi, Atsuko Araki, Sachiko Itoh, Seiko Sasaki, Chihiro Miyashita, Takahiko Mitsui, Hiroyuki Nakazawa, Katsuya Nonomura, Reiko Kishi

**Affiliations:** 1 Center for Environmental and Health Sciences, Hokkaido University, Sapporo, Japan; 2 Department of Public Health, Hokkaido University Graduate School of Medicine, Sapporo, Japan; 3 Department of Urology, Hokkaido University Hospital, Sapporo, Hokkaido, Japan; 4 Department of Analytical Chemistry, Faculty of Pharmaceutical Sciences, Hoshi University, Tokyo, Japan; 5 Department of Renal and Genitourinary Surgery, Graduate School of Medicine, Hokkaido University, Sapporo, Hokkaido, Japan; 6 Kushiro Rosai Hospital, Kushiro, Japan

## Abstract

**Background:**

Perfluorinated chemicals (PFCs) disrupt cholesterol homeostasis. All steroid hormones are derived from cholesterol, and steroid hormones such as glucocorticoids and androgenic hormones mediate several vital physiologic functions. However, the in utero effects of PFCs exposure on the homeostasis of these steroid hormones are not well understood in humans.

**Objectives:**

We examined the relationship between prenatal exposure to perfluorooctane sulfonate (PFOS)/perfluorooctanoate (PFOA) and cord blood levels of glucocorticoid and androgenic hormones.

**Methods:**

We conducted a hospital-based birth cohort study between July 2002 and October 2005 in Sapporo, Japan (n = 514). In total, 185 mother–infant pairs were included in the present study. Prenatal PFOS and PFOA levels in maternal serum samples were measured using liquid chromatography–tandem mass spectrometry (LC-MS-MS). Cord blood levels of glucocorticoid (cortisol and cortisone) and androgenic hormones [dehydroepiandrosterone (DHEA) and androstenedione] were also measured in the same way.

**Results:**

We found a dose–response relationship of prenatal PFOS, but not PFOA, exposure with glucocorticoid levels after adjusting for potential confounders. Cortisol and cortisone concentrations were –23.98-ng/mL (95% CI: –0.47.12, –11.99; p for trend = 0.006) and –63.21-ng/mL (95% CI: –132.56, –26.72; p for trend < 0.001) lower, respectively, in infants with prenatal PFOS exposure in the fourth quartile compared with those in the first quartile. The highest quartile of prenatal PFOS exposure was positively associated with a 1.33-ng/mL higher DHEA level compared with the lowest quartile (95% CI: 0.17, 1.82; p for trend = 0.017), whereas PFOA showed a negative association with DHEA levels (quartile 4 vs. quartile 1: –1.23 ng/mL, 95% CI: –1.72, –0.25; p for trend = 0.004). We observed no significant association between PFCs and androstenedione levels.

**Conclusions:**

Our results indicate that prenatal exposure to PFCs is significantly associated with glucocorticoid and DHEA levels in cord blood.

**Citation:**

Goudarzi H, Araki A, Itoh S, Sasaki S, Miyashita C, Mitsui T, Nakazawa H, Nonomura K, Kishi R. 2017. The association of prenatal exposure to perfluorinated chemicals with glucocorticoid and androgenic hormones in cord blood samples: the Hokkaido Study. Environ Health Perspect 125:111–118; http://dx.doi.org/10.1289/EHP142

## Introduction

Perfluorinated chemicals (PFCs) are humanmade chemicals that have been widely used in different types of industry for the past 60 years. They are ubiquitous and widely detected in the environment, wildlife, and humans. The main pathway of exposure to PFCs in the general population is through the oral route, including digestion of contaminated food and water (D’eon and Mabury 2011; Fromme et al. 2009). Perfluorooctane sulfonate (PFOS) and perfluorooctanoate (PFOA) are the most abundant and commonly detected PFCs in biota and humans. PFOS was added to Annex B of the Stockholm Convention on Persistent Organic Pollutants (POPs) in 2009 (UNEP 2007). Although PFOS and PFOA are being voluntarily phased out by several industries, they are persistent and still present in older products. PFCs are resistant to metabolism and have long elimination half-lives; serum elimination of PFOS and PFOA in human sera is estimated to take 5.4 and 3.8 years, respectively (Olsen et al. 2007). This results in the bioaccumulation of PFCs in the human body.

Recent research has shown that PFCs perturb metabolic end points, including lipid synthesis, glucose metabolism, and thyroid hormone balance in animals (Seacat et al. 2003; Thibodeaux et al. 2003). In addition, PFCs have been shown to decrease testosterone and increased estradiol levels in the serum of mice (Lau et al. 2007; López-Doval et al. 2014; Wan et al. 2011). Some reports have found that PFCs can increase corticosterone in rodents (Austin et al. 2003; Zheng et al. 2009). In contrast, other studies have found that PFCs reduce cortisol and corticosterone levels in salmon and human cells (Liu et al. 2010; Mortensen et al. 2011).

Increased steroid hormone production during pregnancy is essential to meet both the maternal demand for increased estrogens and cortisol production and the fetal demand for reproductive and physical growth and development. In contrast, excess cortisol may also be harmful to the fetus. Pregnancy is a transient physiologic period of hypercortisolism during which cortisol levels increase to two to three times their normal levels. However, the fetus is protected against this cortisol rise, because 11β-hydroxysteroid dehydrogenase 2 (11β-HSD2) in the placenta inactivates cortisol into cortisone. The human fetal adrenal gland is enormous relative to that of the adult organ, and adrenal steroid synthesis is increased in the fetus. The major steroid produced by the fetal adrenal gland is sulfoconjugated dehydroepiandrosterone (DHEAS) and DHEA, which are the main precursors of sex hormones and cortisol antagonists (Mastorakos and Ilias 2003).

A fetus is exposed to PFCs because of maternal–fetal passage during organ development (Inoue et al. 2004). Some epidemiological studies in the general population suggest that these compounds are associated with poor birth outcomes such as reduced birth size (Fei et al. 2007; Johnson et al. 2014; Washino et al. 2009). Cholesterol is a substrate of all steroid hormones. Previous human studies have reported that PFCs may change the cholesterol profile in pregnant (Starling et al. 2014) and nonpregnant women (Frisbee et al. 2010; Winquist and Steenland 2014). Joensen et al. (2013) reported an inverse association between PFOS and testosterone levels in serum samples of adult men. Previously, our group has reported a negative association between prenatal PFOS and progesterone hormone levels of cord blood samples in male and female infants. In addition, PFOS was negatively associated with testosterone/estradiol in male infants, whereas prenatal PFOA exposure was positively associated with progesterone levels in cord blood samples of both sexes (Itoh et al. 2016). However, the effects of PFCs on glucocorticoid hormones and androgenic hormones (the main substrates of testosterone and estrogen) are not well understood in humans.

We investigated whether prenatal exposure to PFOS and PFOA was associated with cortisol and cortisone levels in cord blood samples in a birth cohort using a prospective design. In addition to glucocorticoids, to gain a better understanding of the effects of PFCs on steroidogenesis, we examined the association of PFCs with DHEA and androstenedione as androgenic hormones in cord blood and assessed the balance of glucocorticoids and androgenic hormones in infants.

## Methods

### Study Population

This study was part of the Hokkaido Study on the Environment and Children’s Health conducted between July 2002 and October 2005. The details of this study have been described previously (Kishi et al. 2011, 2013). In this prospective birth cohort, pregnant women between 23 and 35 weeks of gestation were recruited and gave birth at one hospital in Sapporo, Japan. All participants were native Japanese and residents of Sapporo city or the surrounding areas. Of the 1,796 potentially eligible women, the following subjects were excluded: women who decided to participate in the Japanese cord blood bank (22% of those approached) and women who decided to deliver at another hospital (3% of those approached). Ultimately, 514 (28.6%) pregnant women agreed to participate in this study. Of the 514 mother–infant pairs, 10 were excluded due to miscarriage, stillbirth, relocation, or voluntary withdrawal from the study before follow-up.

### Questionnaires and Medical Records

A self-administered questionnaire survey was completed after the second trimester of pregnancy that contained information related to previous medical history, smoking, socioeconomic status, alcohol, and caffeine intake during pregnancy, and food intake frequency during pregnancy including daily fish intake (Washino et al. 2009). A self-administered questionnaire described by Nagata et al. (1998) was used to estimate alcohol and caffeine intake during pregnancy. Medical information, including maternal age, maternal body mass index (BMI) before pregnancy, parity, gestational age, pregnancy complications, type of delivery, infant’s sex, and birth size, was obtained from participant medical records. All participants provided written informed consent and the study protocol was approved by the institutional ethical board for epidemiological studies at the Graduate School of Medicine and the Center for Environmental and Health Sciences, Hokkaido University.

### Blood Sampling and Exposure Assessment

A 40-mL blood sample was taken from the maternal peripheral vein after the second trimester of pregnancy to measure PFOS and PFOA levels. All samples were stored at –80°C until analysis. Detailed methods of the measurement of PFOS and PFOA have been described in our previous report (Goudarzi et al. 2016; Nakata et al. 2009). In brief, serum samples (0.1 mL) were mixed with 0.2 mL internal standard (^13^C_4_-PFOS-Na^+^ and ^13^C_2_-PFOA) solution containing acetonitrile. After centrifugation, the supernatant was transferred to a polypropylene tube. An aliquot of the filtered sample solution was subjected to column-switching liquid chromatography–tandem mass spectrometry (LC-MS/MS). The detection limit for both PFOS and PFOA was 0.5 ng/mL. The PFOS levels were detected in all samples, and for samples with PFOA levels below the detection limit, we used a value of half the detection limit (0.25 ng/mL).

### Outcome Assessment

Cord blood samples (10–30 mL) were collected from the umbilical vein at delivery and stored at –80°C until analysis. Concentrations of cortisol, cortisone, DHEA, and androstenedione were measured in cord blood samples using LC-MS/MS (Yamashita et al. 2007a, 2007b) at Aska Phrama Medical Co., Ltd (Kanagawa, Japan). The detection limits for cortisol and cortisone were 0.250 and 0.100 ng/mL, respectively. The detection limit for DHEA and androstenedione was 0.010 ng/mL.

### Data Analysis

The following subjects were excluded from the analysis of associations between maternal PFCs and glucocorticoids: women with pregnancy-induced hypertension (*n* = 11), women with diabetes mellitus (*n* = 1), mother–infant pairs with fetal heart failure (*n* = 1), and twins (*n* = 7). After exclusion of these subjects, data on PFOS and PFOA concentrations in 429 mother–infant pairs were available. Of those, data on maternal blood during pregnancy and infant cord blood samples in 185 pairs were available and included in the data analysis. Because of the skewed distributions, we treated levels of PFCs and glucocorticoid and androgenic hormones as a continuous variable on a log_10_ scale.

We analyzed correlations between PFOS and PFOA concentrations and the characteristics of the mothers and infants using the Spearman correlation test, the Mann–Whitney *U*-test, and Kruskal–Wallis test. The same statistical analyses were performed to find associations between steroid hormone levels and participants’ characteristics. We performed multiple-regression analysis to examine the association between glucocorticoid and androgenic hormones and the levels of PFCs in maternal serum samples. Potential confounders were selected according to how the covariates in this paper were associated with PFC exposure levels (smoking and caffeine intake during pregnancy, blood sampling period), hormone levels or both (parity) as shown in Tables 1 and 2 (*p* < 0.1). Because of the association of fetal serum steroid hormone levels in humans to gestational age (Rog-Zielinska et al. 2014), we also considered this as a confounder. In addition, we included maternal educational levels as an indicator of socioeconomic status into the fully adjusted models. To assess a dose–response relationship, we divided PFC levels into four quartiles, and least square means (LSMs) and 95% confidence intervals (CI) were calculated. To calculate a value for the trend, we assigned the median concentration to all persons for each corresponding quartile. We compared the least square means of the steroid hormones for each quartile using the Hsu–Dunnet method to accommodate for multiple comparisons. We performed all of the statistical analyses using JMP clinical 5 (SAS Institute Inc., Cary, NC, USA), and results were considered significant when < 0.05.

**Table 1:** Maternal blood PFOS and PFOA levels (ng/mL) in relation to the characteristics of subjects participating in the Hokkaido Study on Environment and Children’s Health, Sapporo, Japan, 2002–2005 (*n* = 185).

Characteristics	*n*	PFOS [mean ± SD, median (25–75 percentile), or correlation]^*a*^	*p*-Value	PFOA [mean ± SD, median (25–75 percentile), or correlation]^*a*^	*p*-Value
Mean ± SD	185	5.78 ± 2.7		1.60 (0.96)
Median (minimum, maximum)	185	5.20 (1.50, 16.20)		1.40 (< LOD, 5.30)
Maternal characteristics
Age (years)^*a*^	185	ρ = –0.047	0.525	ρ = –0.048	0.512
Prepregnancy BMI (kg/m^2^)^*a*^	185	ρ = –0.027	0.712	ρ = –0.031	0.671
Parity^*b*^
0	99	6.37 ± 0.27	0.001	1.94 ± 0.08	< 0.001
≥1	86	5.09 ± 0.29		1.21 ± 0.09
Educational level (years)^*b*^
≤ 12	86	5.59 ± 0.30	0.392	1.53 ± 0.10	0.350
≥ 13	99	5.94 ± 0.27		1.66 ± 0.09
Annual household income (million yen)^*b*^^,^^*c*^
< 5	129	5.64 ± 0.24	0.374	1.63 ± 0.08	0.606
≥ 5	54	6.04 ± 0.37		1.55 ± 0.13
Smoking during pregnancy^*b*^
Yes	33	4.73 ± 0.47	0.015	1.27 ± 0.16	0.027
No	152	6.01 ± 0.22		1.67 ± 0.07
Alcohol intake during pregnancy^*b*^
Yes	60	5.61 ± 0.35	0.324	1.61 ± 0.12	0.904
No	125	5.86 ± 0.24		1.60 ± 0.08
Caffeine intake during pregnancy (mg/day)^*a*^		ρ = –0.083	0.257	ρ = –0.193	0.008
Fish intake during pregnancy^*b*^
Inshore fish
≤ 1–2 times/month	96	5.70 ± 0.28	0.684	1.63 ± 0.09	0.632
≥ 1–2 times/week	89	5.86 ± 0.29		1.57 ± 0.10
Deep sea fish
≤ 1–2 times/month	83	5.60 ± 0.30	0.435	1.59 ± 0.10	0.877
≥ 1–2 times/week	102	5.92 ± 0.27		1.61 ± 0.09
Blood sampling period^*d*^
23–31 weeks	74	6.38 ± 0.30	< 0.001	1.77 ± 0.11	0.086
32–34 weeks	43	6.44 ± 0.40		1.61 ± 0.14
35–41 weeks	68	4.70 ± 0.32		1.41 ± 0.11
Gestational age (days)^*a*^		ρ = 0.028	0.702	ρ = 0.062	0.399
Infant characteristics
Sex^*b*^
Male	81	6.16 ± 0.30	0.100	1.73 ± 0.10	0.117
Female	104	5.48 ± 0.27		1.50 ± 0.09
Birth weight^*a*^	251	ρ = –0.108	0.140	ρ = –0.162	0.026
^***a***^Spearman’s correlation test (ρ). ^***b***^Mann–Whitney *U*-test. ^***c***^Missing data: annual household income (*n* = 2). ^***d***^Kruskal–Wallis test.					

**Table 2:** Cord blood glucocorticoid levels (ng/mL) in relation to characteristics of the subjects participating in the Hokkaido Study on Environment and Children’s Health, Sapporo, Japan, 2002–2005 (*n* = 185).

Characteristics	*n*	Cortisol [mean ± SD or correlation]^*a*^	*p*-Value	Cortisone [mean ± SD or correlation]^*a*^	*p*-Value	DHEA [mean ± SD or correlation]^*a*^	*p*-Value	Androstenedione [mean ± SD or correlation]^*a*^	*p*-Value
Maternal characteristics
Age (years)^*a*^	185	ρ = –0.136	0.063	ρ = –0.134	0.068	ρ = –0.053	0.466	ρ = 0.009	0.894
Prepregnancy BMI (kg/m^2^)^*a*^	185	ρ = 0.044	0.546	ρ = 0.030	0.678	ρ = 0.036	0.626	ρ = 0.020	0.782
Parity^*b*^
0	99	61.2 ± 3.5	< 0.001	108.8 ± 4.1	< 0.001	5.4 ± 1.0	0.266	0.59 ± 0.08	0.477
≥ 1	86	33.4 ± 3.7		82.2 ± 4.4		3.7 ± 1.1		0.68 ± 0.09
Educational level (years)^*b*^
≤ 12	86	48.6 ± 4.0	0.915	94.0 ± 4.6	0.519	5.2 ± 1.1	0.452	0.63 ± 0.09	0.908
≥ 13	99	48.0 ± 3.7		98.2 ± 4.3		4.1 ± 1.0		0.64 ± 0.08
Annual household income (million yen)^*b*^^,^^*c*^
< 5	129	49.9 ± 3.3	0.499	98.0 ± 3.7	0.554	4.2 ± 0.87	0.612	0.62 ± 0.07	0.101
≥ 5	54	45.8 ± 5.1		93.8 ± 5.8		5.0 ± 1.3		0.67 ± 0.11
Smoking during pregnancy^*b*^
Yes	33	40.9 ± 6.5	0.901	95.0 ± 7.5	0.852	3.8 ± 1.7	0.623	0.50 ± 0.14	0.318
No	152	48.1 ± 3.0		96.5 ± 3.5		4.8 ± 0.8		0.66 ± 0.06
Alcohol intake during pregnancy^*b*^
Yes	60	42.8 ± 4.8	0.169	95.0 ± 5.6	0.789	4.0 ± 1.3	0.312	0.70 ± 0.10	0.47
No	125	50.9 ± 3.3		96.8 ± 3.8		4.9 ± 0.9		0.60 ± 0.07
Caffeine intake during pregnancy (mg/day)^*a*^		ρ = –0.004	0.953	ρ = –0.077	0.292	ρ = –0.003	0.966	ρ = –0.063	0.392
Fish intake during pregnancy^*b*^
Inshore fish
≤ 1–2 times/month	96	46.7 ± 3.8	0.545	95.0 ± 4.4	0.68	3.8 ± 1.0	0.280	0.65 ± 0.08	0.732
≥ 1–2 times/week	89	50.0 ± 3.9		97.6 ± 4.6		5.5 ± 1.0		0.61 ± 0.08
Deep sea fish
≤ 1–2 times/month	83	43.5 ± 4.1	0.122	97.0 ± 4.7	0.836	4.0 ± 1.1	0.489	0.62 ± 0.09	0.850
≥ 1–2 times/week	102	52.1 ± 3.7		95.6 ± 4.3		5.1 ± 1.0		0.64 ± 0.08
Gestational age (days)^*a*^	185	ρ = 0.105	0.153	ρ = 0.037	0.611	ρ = 0.009	0.900	ρ = –0.037	0.608
Infant characteristics
Sex^*b*^
Male	81	44.1 ± 4.1	0.18	97.9 ± 4.8	0.637	3.1 ± 1.1	0.085	0.62 ± 0.09	0.799
Female	104	51.5 ± 3.6		94.9 ± 4.2		5.8 ± 1.0		0.65 ± 0.08
Birth weight^*a*^	185	ρ = 0.001	0.983	ρ = 0.087	0.235	ρ = –0.052	0.476	ρ = 0.048	0.515
^***a***^Spearman’s correlation test (ρ). ^***b***^Mann–Whitney U-test. ^***c***^Missing data: annual household income (*n* = 2).									

## Results

In total, 185 mother–infant pairs were included in this study. The average (± SD) age of the mothers at birth was 29.7 ± 4.7 years; 53.5% of mothers were nulliparous (Table 3). Among pregnant women, 17.8% smoked and 32.4% consumed alcohol during pregnancy. Mean (± SD) birth weight was 3130.4 ± 331.6 g, and 43.8% of newborns were boys. PFOS levels were detected in all of the samples; however, PFOA levels were not detected in 11 maternal serum samples (5.9% of participants). The median (minimum, maximum) values of PFOS and PFOA were 5.20 ng/mL (1.50, 16.20 ng/mL) and 1.40 ng/mL (< limit of detection, 5.30 ng/mL), respectively (Table 1). PFOS and PFOA concentrations were modestly correlated (Spearman’s rho = 0.270, *p* < 0.001). We observed statistically significant differences in mean PFOS concentrations by parity, smoking during pregnancy, and blood sampling period. Additionally, there were significant differences in mean PFOA concentrations by parity and smoking and caffeine intake during pregnancy.

**Table 3:** Characteristics of subjects participating in the Hokkaido Study on Environment and Children’s Health, Sapporo, Japan, 2002–2005 (*n* = 185).

Characteristics	*n* (%) or mean ± SD
Maternal characteristics
Age (years)	29.7 ± 4.7
Prepregnancy BMI (kg/m^2^)	21.0 ± 2.9
Parity
0	99 (53.5)
≥1	86 (46.5)
Educational level (years)
*≤ 12*	86 (46.5)
*≥ 13*	99 (53.5)
Annual household income (million yen)^*a*^
< 5	129 (70.5)
≥ 5	54 (29.5)
Smoking during pregnancy
Yes	33 (17.8)
No	152 (82.2)
Alcohol intake during pregnancy
Yes	60 (32.4)
No	125 (67.6)
Caffeine intake during pregnancy (mg/day)	143.4 ± 126.2
Blood sampling period
23–31 weeks	74 (40)
32–34 weeks	43 (23.2)
35–41 weeks	68 (36.8)
Gestational age (days)	278.9 ± 6.7
Infant characteristics
Sex
Male	81 (43.8)
Female	104 (56.2)
Birth weight	3130.4 ± 331.6
Birth length	48.4 ± 1.9
^***a***^Missing data: annual household income (*n* = 2).	

Median (25th–75th percentile) values of cortisol, cortisone, DHEA, and androstenedione in cord blood samples were 39.0 (22.5–65.6), 96.7 (69.1–124.4), 2.3 (1.8–3.1), and 0.45 (0.36–0.58) ng/mL, respectively (Table 4). The detection rate of these steroid hormones in cord blood samples was 100%. We found a strong positive correlation between cortisol and cortisone as well as DHEA and androstenedione levels. Furthermore, glucocorticoids showed a negative correlation with DHEA levels (see Table S1).

**Table 4:** Concentrations (ng/mL) of steroid hormones in cord blood samples (*n* = 185).

Hormone	*n*	Mean ± SD	Median (25th–75th)	> LOD (%)
Cortisol
Total	185	48.3 ± 37.6	9.0 (22.5–65.6)	100
Male	81	44.1 ± 30.6	38.2 (21.1–59.6)	100
Female	104	51.5 ± 42.0	39.7 (24.4–67.0)	100
Cortisone
Total	185	96.2 ± 43.3	96.7 (69.1–124.4)	100
Male	81	97.9 ± 38.7	97.2 (72.4–126.0)	100
Female	104	94.9 ± 46.8	95.2 (66.3–124.5)	100
DHEA
Total	185	4.6 ± 10.2	2.3 (1.8–3.1)	100
Male	81	3.1 ± 4.2	2.1 (1.6–2.7)	100
Female	104	5.8 ± 13.1	2.6 (1.9–3.4)	100
Androstenedione
Total	185	0.63 ± 0.84	0.45 (0.36–0.58)	100
Male	81	0.62 ± 0.72	0.47 (0.36–0.60)	100
Female	104	0.65 ± 0.93	0.44 (0.35–0.57)	100

The relationships between the cord blood levels of steroids and maternal and infant characteristics are shown in Table 2. Cortisol and cortisone levels in cord blood showed a negative association with maternal age (*p* < 0.07). Glucocorticoid levels in cord blood of infants with multiparous mothers were significantly lower compared with those in infants with nulliparous mothers (*p* < 0.001). Gestational age had a nonsignificant positive correlation with cortisol levels. DHEA and androstenedione levels did not show any significant association with maternal or infant characteristics.

As shown in Table 5, after controlling for potential confounders, prenatal PFOS concentration was inversely associated with cortisol levels (β = –0.844; 95% CI: –1.31, –0.378; *p* < 0.001). Similarly, we observed a significant negative association between PFOS and cortisone levels (β = –1.15; 95% CI: –1.79, –0.515; *p* < 0.001). In addition, prenatal PFOS concentrations were positively associated with DHEA levels (β = 0.365; 95% CI: 0.112, 0.618; *p* = 0.004). We found a nonsignificant positive association between PFOA and cortisol (β = 0.244; 95% CI: –0.119, 0.607; *p* = 0.186) and cortisone levels (β = 0.390; 95% CI: –0.108, 0.889; *p* = 0.124). Prenatal exposure to PFOA was negatively associated with DHEA levels (β = –0.250; 95% CI: –0.442, –0.058; *p* = 0.010). In addition, we assessed the association of PFCs with the ratios of cortisol to cortisone, cortisol to DHEA, and glucocorticoid to androgenic hormones. PFOS was negatively associated with the ratios of cortisol/DHEA and glucocorticoid/androgenic hormones but positively associated with cortisol/cortisone ratio. In contrast, PFOA showed a positive nonsignificant association with cortisol to DHEA ratio, and the glucocorticoid to androgenic hormones ratios. For further examination, the association of PFCs with glucocorticoids and androgenic hormones was stratified by sex to identify any sex differences. However, we did not find any specific differences by sex, just that the association of PFCs and DHEA was stronger among boys than among girls (see Tables S2 and S3).

**Table 5:** Association of prenatal PFC levels with cord blood glucocorticoids and androgenic hormones (*n* = 185).

Hormone	PFOS		PFOA	
	β (95% CI)	*p*-Value	β (95% CI)	*p*-Value
Cortisol
Crude	–0.774 (–1.19, –0.356)	< 0.001	0.231 (–0.083, 0.546)	0.149
Adjusted^*a*^	–0.844 (–1.31, –0.378)	< 0.001	0.244 (–0.119, 0.607)	0.186
Cortisone
Crude	–1.11 (–1.68, –0.555)	< 0.001	0.222 (–0.205, 0.650)	0.306
Adjusted^*a*^	–1.15 (–1.79, –0.515)	< 0.001	0.390 (–0.108, 0.889)	0.124
DHEA
Crude	0.342 (0.120, 0.564)	0.002	–0.155 (–0.320, 0.009)	0.064
Adjusted^*a*^	0.365 (0.112, 0.618)	0.004	–0.250 (–0.442, –0.058)	0.010
Androstenedione
Crude	–0.038 (–0.212, 0.134)	0.657	–0.099 (–0.225, 0.026)	0.119
Adjusted^*a*^	–0.013 (–0.208, 0.181)	0.893	–0.105 (–0.251, 0.041)	0.157
Cortisol/cortisone
Crude	0.344 (0.086, 0.602)	0.009	0.008 (–0.183, 0.200)	0.928
Adjusted^*a*^	0.312 (0.025, 0.599)	0.032	–0.146 (–0.364, 0.072)	0.188
Cortisol/DHEA ratio
Crude	–1.11 (–1.71, –0.516)	< 0.001	0.386 (–0.064, 0.838)	0.092
Adjusted^*a*^	–1.21 (–1.88, –0.531)	< 0.001	0.494 (–0.030, 1.02)	0.064
Glucocorticoid/androgenic hormones ratio
Crude	–1.24 (–1.89, –0.608)	< 0.001	0.368 (–0.115, 0.852)	0.134
Adjusted^*a*^	–1.33 (–2.05, –0.601)	< 0.001	0.551 (–0.013, 1.11)	0.057
Both exposure and outcome measures were log_10_ transformed. ^***a***^Fully adjusted for gestational age, maternal age, parity, smoking and caffeine intake during pregnancy, maternal educational level, and blood sampling period.				

**Figure 1:**
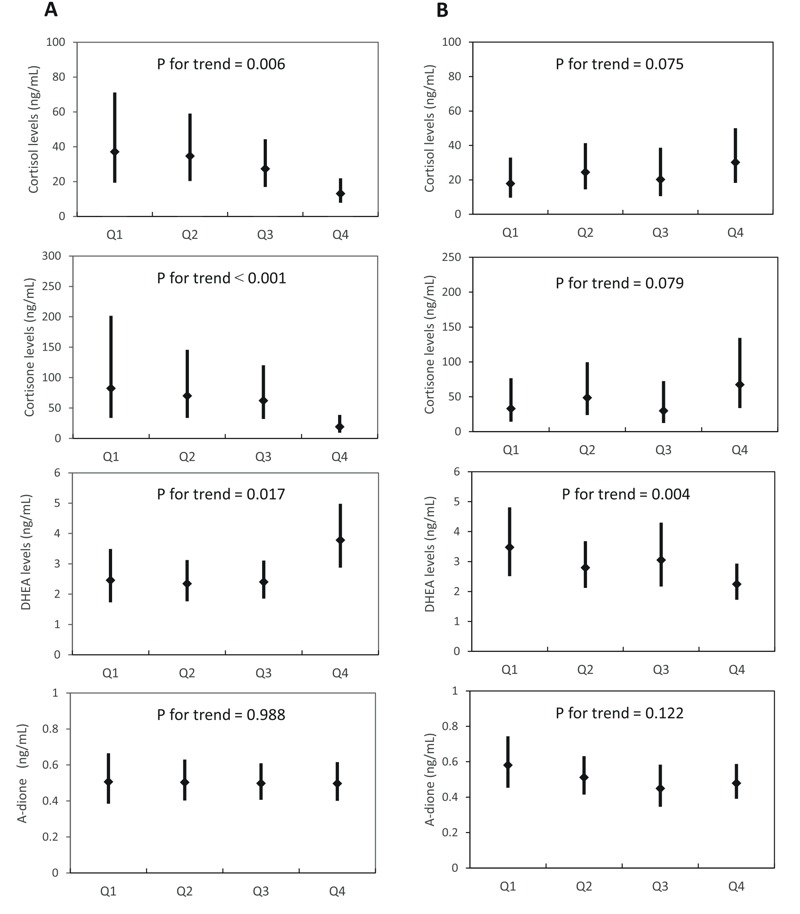
The dose–response relationship of prenatal PFOS (*A*) and PFOA (*B*) quartiles with glucocorticoid and DHEA levels in cord blood, Sapporo, Japan, 2002–2005 (*n* = 185). The LSMs were adjusted for gestational age, maternal age, smoking and caffeine intake during pregnancy, parity, maternal educational level, and the blood sampling period. The LSMs were back transformed from log_10_ to normal values, and the error bars depict the upper and lower 95% CI. Q, quartile.

We also divided maternal PFC levels into quartiles and examined the dose–response relationship between PFCs and steroid hormones (Figure 1; see also Table S4). The quartile analysis after full adjustment showed that the highest quartile of PFOS was associated with a –23.98 ng/mL (95% CI: –47.12, –11.99; *p* for trend = 0.006) in cortisol and –63.21 ng/mL (95% CI: –132.56, –26.72; *p* for trend < 0.001) in cortisone levels compared with the lowest quartile. PFOA showed a positive trend for glucocorticoid levels but was not statistically significant. In addition, we found significant increases in DHEA levels across PFOS quartiles (quartile 4 vs. 1 difference: 1.33 ng/mL; 95% CI: 0.17, 1.82; *p* for trend = 0.017), but significant decreases in DHEA levels among PFOA quartiles (quartile 4 vs. 1 difference = –1.23 ng/mL; 95% CI: –1.72, –0.25; *p* for trend = 0.004). We did not observe a dose–response relationship between PFCs and androstenedione levels.

## Discussion

In this study we addressed the association of PFCs with cord blood glucocorticoid and androgenic hormone levels in a prospective birth cohort. We found a significant negative association of prenatal PFOS levels with cortisol and cortisone levels in cord blood samples. In addition, we found a nonsignificant positive association of prenatal PFOA with cortisol and cortisone levels. We observed a positive association between PFOS and DHEA levels, whereas PFOA was inversely associated with DHEA levels. Our results provide new evidence regarding the association of exposure to low levels of PFCs *in utero* with the concentration of glucocorticoid and androgenic hormones in the next generation.

Median maternal concentrations for PFOS and PFOA in the present study were 5.2 and 1.4 ng/mL, respectively, which are lower than the median values of cohorts conducted in the United States (PFOS: 8.2, PFOA: 2.9 ng/mL) (Stein et al. 2012), Denmark (PFOS: 21.5, PFOA: 3.7 ng/mL) (Halldorsson et al. 2012), Norway (PFOS: 13, PFOA: 2.2 ng/mL) (Starling et al. 2014), and Korea (PFOS: 9.3, PFOA: 2.6 ng/mL) (Lee et al. 2013).

Perinatal steroid hormone concentrations and their variabilities play an essential part in ensuring optimal conditions for the start of human life and maintaining homeostasis in the postnatal period. The correlation between steroid hormones in maternal and cord blood samples is poor. Therefore, steroid hormone concentrations of cord blood provide a better indicator of fetal endocrine milieu (Troisi et al. 2003). Steroid hormones fluctuate during pregnancy and in postnatal life; for example, androgenic hormones gradually increase in maternal blood during pregnancy and are present in high levels at birth in cord blood but rapidly decrease during early infancy (Kuijper et al. 2013). Also, animal and human data suggest that perinatal glucocorticoid levels program the fetal hypothalamic–pituitary–adrenal (HPA) axis affecting its development, resulting in changes in HPA axis function that persist throughout life (Kapoor et al. 2008; Waffarn and Davis 2012). Therefore, cord blood glucocorticoid and androgenic hormone levels may be appropriate indicators to predict HPA axis function and health in later life.

The values we obtained for glucocorticoids in cord blood samples in our study are comparable with those in cord blood samples both in and outside of Japan (Anderson et al. 2010; Hasegawa et al. 2010). In our study, glucocorticoid levels did not differ by sex, and the average DHEA level was higher in female infants, which is in line with previous studies (Ishimoto and Jaffe 2011). Pregnancy is a transient, but physiologic, period of hypercortisolism, and glucocorticoids are essential for regulating and/or modulating normal physiologic functions in metabolism, growth, neurodevelopment, the immune system, blood pressure maintenance, and fluid and electrolyte homeostasis (Braun et al. 2013; Reynolds 2010). Moreover, glucocorticoids have a crucial role in late gestational lung and heart maturation, and insufficient or excess amounts of these hormones have lifelong adverse effects on the cardiovascular system (Ishimoto and Jaffe 2011; Rog-Zielinska et al. 2014). In addition, cord blood cortisol level is lower in infants with intrauterine growth retardation than in infants with appropriate growth for their gestational age (Strinic et al. 2007). However some other reports suggest associations between prenatal exposure to synthetic glucocorticoids and reduced birth size (Khan et al. 2011). Our findings suggest that dyshomeostasis of glucocorticoids and DHEA at birth are associated with *in utero* PFCs exposure, and this may have adverse effects on the HPA axis and steroid hormone homeostasis in later life. Therefore, *in utero* PFC exposure may be a public health concern, and long-term observation of these effects is warranted.

Fetal serum glucocorticoid levels in humans are related to gestational age (Rog-Zielinska et al. 2014). During data analysis, we used gestational age (days) as a confounder in the adjusted model to reduce the possibility of the effect of short gestational age on decreased cortisol and cortisone levels in cord blood. Within our study population, only one infant was premature (gestational age < 37 weeks). For further examination, we excluded this subject from the data analysis, but this did not change the results, indicating that the results may not be biased by gestational age as a determinant factor of glucocorticoid levels. Some previous studies have suggested that glucocorticoid levels in the cord blood of infants born by vaginal delivery are higher than those in infants born by cesarian section (Reissland and Wandinger 1999). In our study, all pregnant women had a vaginal delivery at the same hospital, with uncomplicated singleton pregnancies. Therefore, this cannot be a confounder in our study. Our results showed a significant negative association of glucocorticoids in cord blood with maternal age and multiparity, which is consistent with previous studies (Goedhart et al. 2010). Because of the importance of parity on glucocorticoid levels, we stratified the association between PFOS and glucocorticoids by parity (0, ≥ 1) and observed that the association between PFOS and glucocorticoids was stronger in infants whose mothers were nulliparous. For further assessment of the association between PFCs and glucocorticoids, we included the Apgar score (1 min after birth, continuous variable) as an indicator of infant stress at birth into the adjusted model, and the results remained consistent. Moreover, PFOA and PFOS levels were not strongly correlated in this study (Spearman’s rho = 0.270), and mutual adjustment did not change the results in any consistent way.

The fetal adrenal gland uses large amounts of progesterone supplied by the placenta for cortisol synthesis (Mastorakos and Ilias 2003). PFOS can inhibit the secretion of progesterone in a concentration-dependent manner in human placental syncytiotrophoblasts (Zhang et al. 2015). Barrett et al. (2015) reported a negative association between serum PFOS exposure (but not PFOA) and saliva progesterone levels in healthy nulliparous women 25–35 years of age. In addition, we reported that prenatal exposure to PFOS was inversely associated with progesterone levels in cord blood of male and female infants in the same cohort. In contrast, prenatal PFOA levels were positively associated with cord blood progesterone levels in male and female infants (Itoh et al. 2016). Therefore, this may partly explain the negative association of PFOS but not PFOA with glucocorticoids in the present study. Previously, we reported a negative association between prenatal exposure to PFOS and maternal triglyceride and long-chain polyunsaturated fatty acids (FAs) during pregnancy, including essential FAs and omega 3 and omega 6 FAs in the same cohort (Kishi et al. 2015), whereas PFOA had a nonsignificant positive association with triglyceride and most FAs. However, we did not measure cholesterol levels in this population, making it difficult to interpret the mechanistic effects of PFCs on steroid hormones through cholesterol metabolism.

The mode of action(s) that explains the correlation between PFC exposure and steroid hormones is not fully understood. However, the most putative targets of PFCs are nuclear receptors including peroxisome proliferator-activated receptor (PPAR)α, PPARγ, estrogen, and androgen receptors (Kjeldsen and Bonefeld-Jørgensen 2013; Takacs and Abbott 2007; Vanden Heuvel et al. 2006). These genes are involved in cholesterol metabolism, lipid transport, and steroid synthesis. Therefore, PFCs may change steroid hormone homeostasis by affecting different genes and mechanisms of action.

PFCs, especially PFOS, inhibit the activity of several enzymes in the pathway of steroidogenesis in human cells, such as 3β-hydroxysteroid dehydrogenase (HSD3B), that convert pregnenolone to progesterone and DHEA to androstenedione (Zhao et al. 2010). PFOS, and PFOA with weaker potency, inhibits 11β-hydroxysteroid dehydrogenase 2 (11β-HSD2) which converts cortisol to cortisone (Ye et al. 2014; Zhao et al. 2011). Therefore, these modified enzyme activities may disrupt the ratios of cortisol/cortisone, cortisol/DHEA, and C19-steroids (androgenic hormones) and C21-steroids (glucocorticoids). We found that PFOS is associated with a decrease in the cortisol/DHEA ratio and glucocorticoid/androgenic hormone ratio, indicating that PFOS may shift steroidogenesis to androgenic hormones (Table 5). Also, PFOS was associated with an increased cortisol/cortisone ratio, which suggests inhibition of 11β-HSD2 enzyme. In contrast, PFOA was associated with these ratios in the opposite direction. In this study, we found that the direction of PFOS and PFOA effects on steroids are different, and further studies are necessary to replicate these findings and clarify the mechanistic effects of these PFCs on steroidogenesis.

In this study, we measured prenatal PFC levels in a prospective birth cohort and have provided new evidence regarding the association between *in utero* PFC exposure levels and cord blood steroid hormones. In addition, we assessed steroid hormone levels using LC-MS/MS, which has a very high sensitivity and specificity compared with immunoassay. However, this study has some limitations. The participation rate in this study was low due to the exclusion of eligible women who decided to participate in the Japanese cord blood bank. In addition, only mother–infant pairs with available prenatal and cord blood samples (*n* = 185) were included in the present study, which may have led to potential selection bias. Mother–infants pairs included in the present analysis, compared with the original cohort, were more often primipara, had longer gestational age, and had a lower percentage of male infants (see Table S5). However, compared with the original cohort, mother–infant pairs in the present data analysis had similar PFCs exposure levels as well as characteristics including maternal age, prepregnancy maternal BMI, socioeconomic status, and smoking rate during pregnancy.

Our group previously reported time trends of 11 types of PFCs between 2003 and 2011 in plasma samples during pregnancy in Hokkaido, Japan. The results indicated that concentrations of PFOS and PFOA were declining, whereas levels of PFCs with longer carbon chains such as PFNA and PFDA were increasing (Okada et al. 2013). Additionally, previous laboratory studies suggest greater toxicity of PFCs with longer carbon chains (Wolf et al. 2008). Therefore, more studies to clarify the effects of PFCs with longer carbon chains on steroid hormone profile are necessary. In addition, some recent studies have suggested that the placenta plays an important role in regulation of 11β-HSD2 enzyme activity and glucocorticoid levels resulting in poor birth weight and neurodevelopment outcomes (Marsit et al. 2012; Waffarn and Davis 2012). The placenta is an important tissue for regulating endogenous hormone synthesis and passage. Examination of placental tissue along with cord blood may be a promising approach for future studies.

## Supplemental Material

(223 KB) PDF
