# Supplementary material for: The Association of Prenatal Exposure to Perfluorinated Chemicals with Glucocorticoid and Androgenic Hormones in Cord Blood Samples: The Hokkaido Study
Source: Environ Health Perspect. 2016 May 24;125(1):111–8. doi: 10.1289/EHP142 (PMC5226690; doi:10.1289/EHP142)
Supplement: (223 KB) PDF [file EHP142.s001.acco.pdf]

Environ Health Perspect
DOI: 10.1289/EHP142

Note to readers with disabilities: *EHP* strives to ensure that all journal content is accessible to all readers. However, some figures and Supplemental Material published in *EHP* articles may not conform to 508 standards due to the complexity of the information being presented. If you need assistance accessing journal content, please contact ehp508@niehs.nih.gov. Our staff will work with you to assess and meet your accessibility needs within 3 working days.

Supplemental Material

The Association of Prenatal Exposure to Perfluorinated Chemicals with Glucocorticoid and Androgenic Hormones in Cord Blood Samples: The Hokkaido Study

Houman Goudarzi, Atsuko Araki, Sachiko Itoh, Seiko Sasaki, Chihiro Miyashita, Takahiko Mitsui, Hiroyuki Nakazawa, Katsuya Nonomura, and Reiko Kishi

Table of Contents

Table S1. Correlations between cord blood glucocorticoid and androgenic hormones (n=185)
Table S2. Association of prenatal PFC levels and cord blood cortisol and cortisone by sex stratification.
Table S3. Association of prenatal PFC levels with cord blood DHEA, and androstenedione by sex stratification.
Table S4. Adjusted least square means (LSM) and regression coefficients (β) for glucocorticoid and androgenic hormones in cord blood samples by quartiles of PFCs (n=185).
Table S5. Characteristics of mother-infant pairs in current analysis (n=185) compare with non-participants in this analysis (n=244) and participants in original cohort (n=429).

Table S1. Correlations between cord blood glucocorticoid and androgenic hormones (n=185)

	Cortisol		Cortisone		DHEA		Androstene-dione	
	ρ*	p-value	ρ	p-value	ρ	p-value	ρ	p-value
Cortisol	1							
Cortisone	0.641	<0.001	1					
DHEA	-0.204	0.005	-0.301	<0.001	1			
Androstenedione	0.113	0.123	0.154	0.035	0.329	<0.001	1	

* ρ: Spearman Rho

Table S2. Association of prenatal PFC levels and cord blood cortisol and cortisone by sex stratification.

	PFOS			PFOA		
	β	(95% CI)	p-value	β	(95% CI)	p-value
Boys (n=81)						
Cortisol						
Crude	-0.237	(-1.27, -0.056)	0.032	0.141	(-0.176, 0.803)	0.206
Adjusted[a]	-0.262	(-1.39, -0.071)	0.030	0.195	(-0.177, 1.04)	0.162
Cortisone						
Crude	-0.308	(-1.90, -0.349)	0.005	0.084	(-0.400, 0.890)	0.452
Adjusted[a]	-0.291	(-1.92, -0.204)	0.016	0.210	(-0.164, 1.42)	0.118
Girls (n=104)						
Cortisol						
Crude	-0.279	(-1.46, -0.284)	0.004	0.088	(-0.234, 0.624)	0.369
Adjusted[a]	-0.326	(-1.76, -0.282)	0.007	0.095	(-0.280, 0.700)	0.397
Cortisone						
Crude	-0.283	(-2.02, -0.409)	0.003	0.055	(-0.422, 0.757)	0.574
Adjusted[a]	-0.309	(-2.36, -0.289)	0.012	0.104	(-0.368, 0.998)	0.362

[a] Fully adjusted for gestational age, maternal age, parity, smoking and caffeine intake during pregnancy, maternal educational level, and blood sampling period.
Both exposure and outcome measures were $\log_{10}$ transformed.

Table S3. Association of prenatal PFC levels with cord blood DHEA, and androstenedione by sex stratification.

	PFOS			PFOA		
	β	(95% CI)	p-value	β	(95% CI)	p-value
Boys (n=81)						
DHEA						
Crude	0.290	(0.106, 0.701)	0.008	-0.148	(-0.406, 0.080)	0.185
Adjusted [a]	0.308	(0.099, 0.755)	0.011	-0.312	(-0.642, -0.043)	0.025
Androstenedione						
Crude	-0.092	(-0.390, 0.161)	0.410	-0.193	(-0.404, 0.026)	0.084
Adjusted [a]	-0.011	(-0.312, 0.284)	0.926	-0.230	(-0.490, 0.038)	0.093
Girls (n=104)						
DHEA						
Crude	0.232	(0.068, 0.694)	0.017	-0.091	(-0.329, 0.199)	0.354
Adjusted [a]	0.238	(-0.016, 0.796)	0.059	-0.170	(-0.457, 0.065)	0.140
Androstenedione						
Crude	0.004	(-0.224, 0.236)	0.960	-0.073	(-0.221, 0.100)	0.460
Adjusted [a]	0.004	(-0.290, 0.300)	0.972	-0.096	(-0.267, 0.109)	0.406

[a] Fully adjusted for gestational age, maternal age, parity, smoking and caffeine intake during pregnancy, maternal educational level, and blood sampling period.

Both exposure and outcome measures were log_{10} transformed.

Table S4. Adjusted [a] least square means (LSM) and regression coefficients (β) for glucocorticoid and androgenic hormones in cord blood samples by quartiles of PFCs (n=185).

	Adjusted[a]	Cortisol			Adjusted[a]	Cortisone			Adjusted[a]	DHEA			Adjusted[a]	Androstenedione		
	LSM	β	LCI	UCI	LSM	β	LCI	UCI	LSM	β	LCI	UCI	LSM	β	LCI	UCI
PFOS in quartiles (ng/ml)																
Quartile 1 (1.50-3.85)	37.12	Reference			82.48	Reference			2.46	Reference			0.51	Reference		
Quartile 2 (3.85-5.20)	34.66	-2.46	-30.29	12.49	70.09	-12.39	-94.96	32.04	2.35	-0.11	-0.55	0.73	0.50	0.00	-0.09	0.12
Quartile 3 (5.20-7.25)	27.39	-9.73	-34.31	6.41	62.16	-20.32	-94.17	34.87	2.40	-0.06	-0.63	0.63	0.50	-0.01	-0.09	0.12
Quartile 4 (7.25-16.2)	13.14	-23.98	-47.12	-11.99	19.27	-63.21	-132.56	-26.72	3.78	1.33	0.17	1.82	0.50	-0.01	-0.12	0.09
p for trend	0.006				<0.001				0.017				0.988			
Q1 vs Q4 (p-value)	0.007				0.006				0.048				0.998			
PFOA in quartiles (ng/ml)																
Quartile 1 (<LOD-0.90)	17.85	Reference			33.12	Reference			3.48	Reference			0.58	Reference		
Quartile 2 (0.90-1.40)	24.51	6.66	-7.86	26.99	48.69	15.57	-23.96	74.84	2.79	-0.68	-1.31	0.17	0.51	-0.07	-0.16	0.05
Quartile 3 (1.40-2.20)	20.24	2.39	-16.85	16.43	29.82	-3.30	-46.26	41.97	3.05	-0.43	-1.26	0.40	0.45	-0.13	-0.20	0.03
Quartile 4 (2.20-5.30)	30.24	12.39	-7.23	34.41	67.43	34.32	-19.97	110.04	2.25	-1.23	-1.72	-0.25	0.48	-0.10	-0.19	0.03
p for trend	0.075				0.079				0.004				0.122			
Q1 vs Q4 (p-value)	0.272				0.284				0.042				0.350			

[a] Fully adjusted for gestational age, maternal age, parity, smoking and caffeine intake during pregnancy, maternal educational level, and blood sampling period.
LOD: limit of detection

Table S5. Characteristics of mother-infant pairs in current analysis (n=185) compare with non-participants in this analysis (n=244) and participants in original cohort (n=429).

	Total (n=429)	Non-participants (n=244)	Participants (n= 185)
Characteristics	No. (%)/<br>mean ± SD	No. (%)/<br>mean ± SD	No. (%)/<br>mean ± SD
PFOS (median, 25-75 percentile)	5.2 (3.4-7.0)	5.2 (3.2-6.9)	5.2 (3.8-7.2)
PFOA (median, 25-75 percentile)	1.3 (0.8-1.8)	1.2 (0.7-1.6)	1.4 (0.9-2.2)
**Maternal characteristics**			
Age (years)	30.2 ± 4.7	30.5±4.8	29.7±4.7
Prepregnancy BMI (kg/m^2)	21.1 ± 3.0	21.2±3.2	21.0±2.9
Parity; primipara	202 (47.1)	103 (42.4)	99 (53.5)
Maternal educational level; ≤12 years)	192 (44.7)	106 (43.4)	86 (46.5)
Annual income during pregnancy; <5 million yen	296 (69.4)	167 (68.7)	129 (70.5)
Smoking during pregnancy; yes	73 (17.0)	40 (16.4)	33 (17.8)
Alcohol intake during pregnancy; yes	129 (30.0)	69 (28.2)	60 (32.4)
**Child characteristics**			
Male infants	198 (46.1)	117 (47.9)	81 (43.8)
Gestational age (days)	275.4 ± 10.0	272.7±11.1	278.9±6.7
